# Effect of delayed timing of artificial insemination with sex-sorted semen on pregnancy per artificial insemination in synchronized dairy heifers managed in a seasonal-calving pasture-based system

**DOI:** 10.3168/jdsc.2022-0363

**Published:** 2023-07-21

**Authors:** S.G. Moore, A.D. Crowe, F. Randi, S.T. Butler

**Affiliations:** a Animal & Grassland Research and Innovation Centre, Teagasc, Moorepark, Fermoy, Co. Cork, Ireland P61 P302; b School of Agriculture and Food Science, University College Dublin, Belfield, Dublin 4, Ireland D04 N2E5; c Ceva Santé Animale, Libourne, Bordeaux, France 33500

## Abstract

The objective of this study was to evaluate the timing of artificial insemination (AI) with frozen-thawed sex-sorted semen on pregnancy per AI (P/AI) in dairy heifers. A 6-d progesterone Co-Synch protocol was used for ovulation synchronization of dairy heifers, with timed AI (TAI) coincident with (TAI-0) or 8 h (TAI-8) after the second injection of GnRH, corresponding to either 48 h or 56 h after removal of the progesterone-releasing intravaginal device. Pregnancy diagnosis was conducted by transrectal ultrasound scanning of the uterus 34 d after TAI (n = 816 records available for analysis). Generalized linear mixed models were used to examine the effects of treatment on P/AI. Treatment (n = 2), herd (n = 11), and treatment × herd were included as categorical fixed effects. Heifer body weight and Economic Breeding Index values for milk production, fertility, calving performance, beef carcass, cow maintenance, cow management, and health were included as continuous fixed effects. Heifer ID was included as a random effect. Pregnancy per AI was greater for TAI-8 heifers (59%) compared with TAI-0 heifers (50%). Pregnancy per AI ranged from 38% to 75% between herds but there was no treatment × herd interaction. The fertility subindex (positive) and the cow management subindex (negative) were the only continuous animal variables associated with P/AI. Delaying the timing of AI with frozen-thawed sex-sorted semen by 8 h in dairy heifers enrolled on a 6-d progesterone Co-Synch protocol improved P/AI.

## Highlights

• Pregnancy rates in heifers improved by delaying the timing of artificial insemination with sexed semen by 8 hours.
• Good pregnancy rates were observed in heifers inseminated following a 6-day progesterone Co-Synch protocol.
• Genetic merit for cow fertility is positively associated with heifer pregnancy rate.
• Genetic merit for cow management is negatively associated with heifer pregnancy rate.
• Body weight was not associated with heifer pregnancy rate.

## Graphical Abstract

**Figure:**
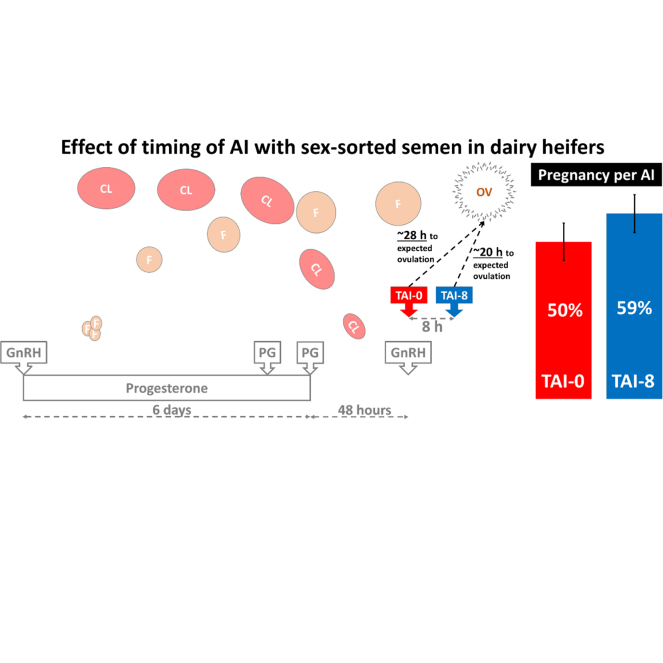
**Summary:** This study evaluated the timing of artificial insemination (AI) with frozen-thawed sex-sorted semen on pregnancy per AI in dairy heifers. A 6-day progesterone Co-Synch protocol was used for ovulation synchronization of 823 dairy heifers, with timed AI (TAI) coincident with (TAI-0) or 8 hours (TAI-8) after the second injection of gonadotropin-releasing hormone (GnRH), corresponding to either 48 or 56 hours after removal of the progesterone-releasing intravaginal device. We hypothesized that TAI-8 heifers would have greater pregnancy rates than TAI-0 heifers by delivering viable sex-sorted semen to the uterus closer to the time of ovulation. The study demonstrated that delaying the normal timing of AI of dairy heifers with frozenthawed sex-sorted semen by 8 hours, thereby delivering sperm cells closer to the presumed time of ovulation, improved pregnancy per AI.

The use of sex-sorted (**SS**) semen to generate dairy replacement heifers is growing rapidly as dairy producers seek to accelerate genetic gain, reduce dystocia in their herds, and produce fewer low-value male dairy calves (Holden and Butler, 2018; Balzani et al., 2021; Creutzinger et al., 2021). Targeted use of SS semen on the dams with the best genetic merit also increases the proportion of the herd on which beef-breed semen can be used to increase the economic value of beef produced from the dairy herd (Holden and Butler, 2018). As the use of SS semen is associated with reduced pregnancy rates compared with using conventional sperm (Maicas et al., 2019; Drake et al., 2020; Maicas et al., 2020), the compactness of the seasonal calving pattern may deteriorate when SS semen is used inappropriately (Ruelle et al., 2021). Sex-sorted semen is therefore prioritized for use on dairy heifers because their fertility and their genetic merit is greater than older lactating cows (Maicas et al., 2019). Combining the use of SS semen with timed AI (**TAI**) to achieve 100% submission rate for eligible animals on the first day of the seasonal breeding period can mitigate any reduction in pregnancy per artificial insemination (**P/AI**), thereby maintaining or even improving the compactness of the seasonal calving pattern (Ruelle et al., 2021).

The longevity of frozen-thawed SS semen is shorter compared with conventional semen, likely due to changes in sperm membranes induced by the sorting process and cryopreservation that accelerates capacitation and acrosome reaction processes (Mocé et al., 2006; de Oliveira Carvalho et al., 2018). Hence, pregnancy rates have been reported to be greater when heifers were inseminated with SS semen at later time points relative to the onset of estrus, likely reflecting the presence of greater numbers of viable sperm cells in the oviduct when the oocyte arrives at the ampulla for fertilization (Vishwanath and Moreno, 2018). In support of this, delaying the timing of AI with SS semen relative to the onset of estrus (i.e., closer to the time of ovulation) was associated with improved pregnancy rates in heifers (Sá Filho et al., 2010).

The 5-d timed AI protocol, with a progesterone (**P4**) device inserted for 5 d, is well established (Rabaglino et al., 2010; Lima et al., 2013), but the efficacy of this protocol for TAI is not optimal because removal of the intravaginal P4 device on d 5 coincident with the administration of the first injection of PGF_2α_ results in ~30% of heifers displaying estrus within the following 36 to 48 h, requiring AI 12 to 24 h before the scheduled TAI (Chebel and Cunha, 2020; Lauber et al., 2021; Macmillan et al., 2021). This situation increases the labor associated with breeding heifers but can be overcome by leaving the intravaginal P4 device in situ for an additional 24 h until d 6, which prevents estrus on the day before scheduled TAI (Lauber et al., 2021). On the other hand, because no heifers express estrus ahead of the scheduled TAI, the timing of AI to be coincident with exogenous GnRH may be too early relative to ovulation for a large proportion of heifers that require that exogenous GnRH before initiating a LH surge to trigger ovulation.

The objective of the current study, therefore, was to compare P/AI in dairy heifers enrolled on a 6-d P4 Co-Synch protocol and inseminated with frozen-thawed SS semen coincident with the second GnRH injection [**TAI-0**; 48 h after progesterone-releasing intravaginal device (**PRID**) removal] or 8 h after the second GnRH injection (**TAI-8**; 56 h after PRID removal). We tested the hypothesis that TAI-8 heifers would have greater pregnancy rates than TAI-0 heifers by delivering viable SS semen to the uterus closer to the time of ovulation.

The study was approved by the Teagasc Animal Ethics Committee (Fermoy, Cork, Ireland). The study took place on 4 research herds operated by Teagasc Moorepark, Fermoy, Co. Cork, Ireland, during April 2021 and on the same 4 herds and an additional 3 commercial herds located near Fermoy, Co. Cork, Ireland, during April 2022. Using power of 80% and a significance level of 0.05, 770 heifers (n = 385 per treatment) were required to detect an increase in P/AI from 50% in TAI-0 treatment to 60% in TAI-8. A 10 percentage point difference in treatment means was deemed by the authors to be a sufficient difference between treatments to merit adoption of one protocol over the other by heifer breeders. In total, 823 heifers not previously submitted for AI were enrolled. In 2021, 263 heifers were enrolled (n = 107, 82, 35, and 39 on research farm 1, 2, 3, and 4, respectively) and in 2022, 560 heifers were enrolled (n = 127, 76, 34, 38, 139, 87, and 59 on research farm 1, 2, 3, 4, and commercial farm 1, 2, and 3, respectively). All heifers in each herd were enrolled on the protocol. One month before breeding, the heifers weighed 294.7 ± 38.5 kg. On the day of timed AI, the heifers were 14.7 ± 0.4 mo of age. Heifers were enrolled on a 6-d P4-Co-Synch protocol (Figure 1). On d −8 (i.e., 8 d before TAI date), a 2-mL i.m. injection of GnRH analog (Ovarelin, 100 µg of gonadorelin diacetate tetrahydrate; Ceva Santé Animale) was administered, and a PRID containing 1.55 g of P4 (PRID Delta; Ceva Santé Animale) was inserted in the vagina. On d −3, a 5-mL i.m. injection of PGF_2α_ (Enzaprost, 25 mg of dinoprost trometamol; Ceva Santé Animale) was administered. On d −2, a second 5-mL i.m. injection of PGF_2α_ was administered and the PRID was removed. On d 0 (48 h after the final PGF_2α_ injection and PRID removal), a second i.m. injection of GnRH was administered.

**Figure 1:**
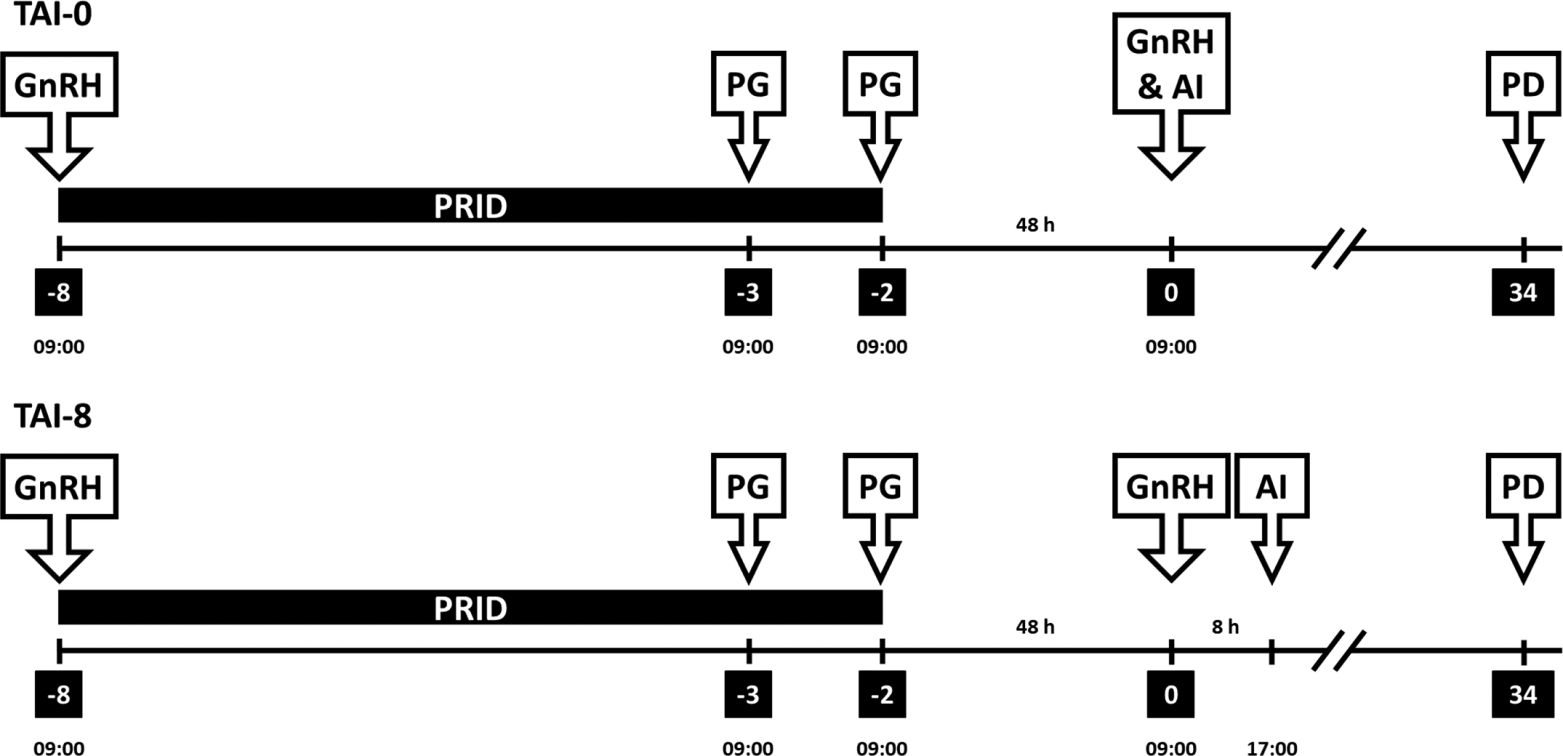
Schematic diagram summarizing the synchronization protocol used and timing of AI. On d −8 (i.e., 8 d before AI date), an injection of GnRH was administered, and a progesterone-releasing intravaginal device (PRID) was inserted. On d −3, an injection of PGF_2α_ (PGF) was administered. On d −2, a second injection of PGF was administered and the PRID was removed at 0900 h. On d 0, an injection of GnRH was administered to all heifers at 0900 h, and timed AI (TAI) was conducted at either 0900 h (coincident with the second GnRH; TAI-0) or 8 h later at 1700 h (8 h after the second GnRH; TAI-8). Pregnancy diagnosis (PD) was conducted using transrectal ultrasound at 34 d after TAI.

The experiment was a completely randomized block design. Within each herd, synchronized heifers were stratified based on breed (Holstein-Friesian, Jersey, Holstein-Friesian × Jersey) and BW, and randomly assigned to 1 of 2 experimental treatments: TAI coincident with the second GnRH injection (TAI-0; n = 410), or TAI 8 h after the second GnRH injection (TAI-8; n = 413). One of 2 different oil-based tail paint colors (Tell Tail, FIL) that corresponded to the heifer treatment was used to make a visible strip across the middle of the back of every heifer on the day of synchronization protocol initiation. This served to aid identification of heifers that required AI coincident with the second GnRH injection (TAI-0) or 8 h after the second GnRH injection (TAI-8). The initiation of the protocol on d −8 was implemented by the research team, and the remaining interventions were undertaken by the herd owner or farm manager. On the day of protocol initiation, the herd owner or farm manager was given their farm-specific timetable of the remaining interventions and the exact supply of required hormones, syringes, and needles. Each AI straw contained 4 × 10^6^ sperm that were processed using the SexedULTRA Genesis III system as described by Vishwanath and Moreno (2018), and sourced from 1 of 3 bovine semen suppliers. All inseminations were completed by professional AI technicians and recorded on handheld devices, and data were subsequently uploaded via an application program interface link to the Irish Cattle Breeding Federation database (www.icbf.com). Upon completion of all inseminations, data including heifer ID number and AI sire code were extracted from the Irish Cattle Breeding Federation database. Sire mate selection was determined by the herd owner or farm manager. Pregnancy was diagnosed by transrectal ultrasound scanning of the uterus 34 d after AI. All pregnancy diagnosis results were compiled into a single database and merged with insemination and heifer information data. Data from 7 heifers that had lost their PRID before the day of its scheduled removal were excluded from the data set, leaving data from a total of 816 heifers (TAI-0: n = 406; TAI-8: n = 410) available for statistical analysis. The average number of heifers per herd was 74 (range 32–139). Semen from 37 different sires was used for the first AI across all herds with an average of 22 inseminations per sire (range 1–94). Of the 37 sires, 29 were used for ≥10 inseminations. Within each farm, use of individual sires was approximately equal at TAI-0 and TAI-8.

Data handling and statistical analysis was performed using SAS 9.4 (SAS Institute Inc.). Data were statistically analyzed using the GLIMMIX procedure of SAS. Treatment (n = 2), herd (n = 11), and treatment × herd were included as categorical fixed effects. Body weight (1 mo before mating start date) and Economic Breeding Index values for milk production, fertility, calving performance, beef carcass, cow maintenance, cow management, and health (obtained from www.icbf.com) were included as continuous fixed effects. Heifer ID was included as a random effect. The binary distribution and logit function were specified.

Pregnancy per AI is reported as LSM with 95% confidence interval in parentheses. There were effects of treatment (*P* = 0.048) and herd (*P* = 0.002) on P/AI, but a treatment × herd interaction effect was not detected (*P* = 0.65). Pregnancy per AI was 8.7 percentage points greater for heifers assigned to TAI-8 [59.1% (52.7%–65.1%)] compared with heifers assigned to TAI-0 [50.4% (44.4%–56.3%)].

The average P/AI across herds was 54.5% and ranged from 38.2% in herd 9 to 75.2% in herd 11 (Figure 2). Of the continuous variables, the fertility subindex (*P* = 0.04) and the management subindex (*P* = 0.009) were associated with P/AI. A 1 unit change in the fertility subindex was associated with 0.006 unit increase in the P/AI such that the predicted increase in P/AI for the range in fertility subindex (€9.5 to €241.1) that we observed was 1.39%. A 1 unit change in the cow management subindex was associated with a 0.945 unit decrease in P/AI such that the predicted decrease in P/AI for the range in the cow management subindex (−€11.8 to €15.0) that we observed was 1.61%.

**Figure 2:**
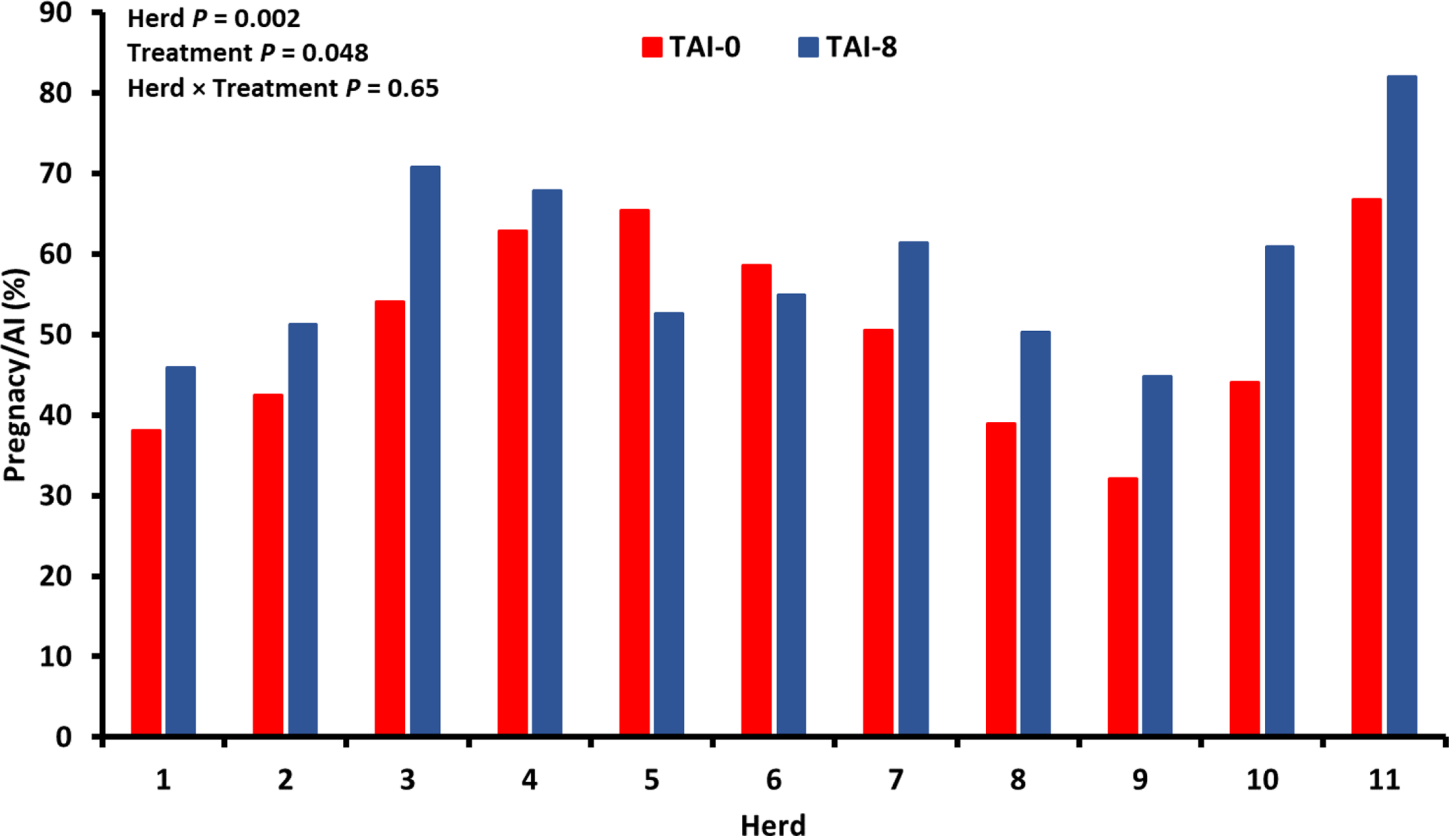
Pregnancy per AI for heifers receiving AI 48 h (TAI-0) and 56 h (TAI-8) after the final PGF_2α_ injection and progesterone-releasing intravaginal device (PRID) removal. Pregnancy per AI was greater in TAI-8 heifers compared with TAI-0 heifers. Data are presented as LSM with 95% CI.

In support of our hypothesis, P/AI was improved in the TAI-8 heifers compared with the TAI-0 heifers. The results also support the previous reports from Sá Filho et al. (2010), Sales et al. (2011), and Bombardelli et al. (2016). In contrast, other studies reported no benefit of delaying the timing of AI on pregnancy rate (72 vs. 84 h after P4 device removal; Chebel and Cunha, 2020), or a tendency to decrease pregnancy rate from 88% to 70% (72 vs. 80 h after P4 device removal; Macmillan et al., 2021). In the study by Lauber et al. (2021), the P4 device remained in the vagina for 5 or 6 d, which corresponded with the P4 device being removed 72 h or 48 h before AI, and P/AI were 49% and 45%, respectively. Lauber et al. (2021) speculated that AI 48 h after P4 device removal was too early and both Chebel and Cunha (2020) and Macmillan et al. (2021) speculated that AI at 80 to 84 h after P4 device removal was too late. The results presented in the current study suggest that 56 h after removal of the P4 device (8 h after the second GnRH) was a better time to AI heifers with frozen-thawed SS semen compared with 48 h after removal of the P4 device (coincident with the second GnRH) when a 6-d P4 Co-Synch protocol is implemented. We presume that the improvement in P/AI for TAI-8 reflects the beneficial effect of delivering SS semen closer to the time of ovulation. It is yet to be determined if a similar benefit to delaying the timing of AI exists when fresh SS semen that has not experienced the freeze-thaw process is used.

The P/AI observed in the heifers assigned to the TAI-8 treatment was 59%, which represents an improvement compared with the figure of 53% previously reported by Maicas et al. (2019) in the last SS semen field trial with heifers in Irish dairy herds. Most of the heifers in the trial reported by Maicas et al. (2019) were inseminated with SS semen (2.1 million sperm cells per straw) following observed estrus without use of a TAI protocol. A feature of the current study was that leaving the P4 device in situ for 6 d instead of the traditional 5 d prevented premature onset of estrus, and thereby avoided the requirement to AI a subset of heifers on the day before scheduled TAI as previously reported by Lauber et al. (2021). Since the purpose of the study was to AI 100% of heifers on the scheduled date regardless of estrus, the estrus status of the heifers was not recorded, although Lauber et al. (2021) reported that 73% of heifers synchronized with the P4 device in situ for 6 d were in estrus 2 d later on the day of scheduled AI.

The variation in P/AI across herds in the current study was considerable, but was similar to that previously reported by Drake et al. (2020) between 24 herds of lactating dairy cows inseminated with SS semen. Nonetheless, explanatory variables associated with the variation in P/AI between herds in this study were not identified. Heifer BW at breeding is an important indicator of subsequent fertility (Archbold et al., 2012); however, heifer BW was not associated with P/AI in the current study. Genetic merit for fertility was positively associated with P/AI, a finding supported by numerous studies with cows and heifers (Cummins et al., 2012; Veronese et al., 2019; Meier et al., 2021). Genetic merit for cow management was negatively associated with P/AI. Temperament is a component trait of the cow management sub-index of the Economic Breeding Index (www.icbf.com). While the phenotypic temperament of the heifers in this study was not recorded, it was previously reported that P/AI was poorer in beef cows and heifers with aggressive or excitable temperaments compared with animals with calm temperaments (Cooke et al., 2012; Kasimanickam et al., 2018).

In summary, this study demonstrated that delaying the normal timing of AI of heifers with frozen-thawed SS semen by 8 h, thereby delivering sperm cells closer to the presumed time of ovulation, improved P/AI. Studies to date, including the current study, have evaluated the time of AI using frozen-thawed SS semen; however, similar studies using fresh SS semen are warranted.
